# How should we evaluate sweetened beverage tax policies? A review of worldwide experience

**DOI:** 10.1186/s12889-021-11984-2

**Published:** 2021-10-26

**Authors:** Shu Wen Ng, M. Arantxa Colchero, Martin White

**Affiliations:** 1 Carolina Population Center, University of North Carolina at Chapel Hill, CB #8120, 137 East Franklin Street, Chapel Hill, NC 27516 USA; 2 Department of Nutrition, Gillings School of Global Public Health, University of North Carolina at Chapel Hill, CB#7461, UNC-Chapel Hill, Chapel Hill, NC 27599-7461 USA; 3 Center for Health Systems Research, Instituto Nacional de Salud Pública, Universidad No 655 Colonia Santa María Ahuacatitlán, Cerrada Los Pinos y Caminera, CP, 62100 Cuernavaca, Morelos Mexico; 4 Centre for Diet and Activity Research, MRC Epidemiology Unit, University of Cambridge, Cambridge, UK

**Keywords:** Sugar-sweetened beverage, Tax, Evaluation, Policy, Logic models

## Abstract

Over 45 jurisdictions globally have implemented sweetened beverage taxes. Researchers and policymakers need to assess whether and how these taxes change beverage demand and supply, their intended and unanticipated health, economic and equity impacts. Lessons from such evaluations can maximise the policies’ success and impact on non-communicable disease prevention globally. We discuss key theoretical, design and methodological considerations to help policymakers, funders and researchers commission and conduct rigorous evaluations of these policies and related disease prevention efforts. We encourage involving the perspectives of various stakeholders on what evaluations are needed given the specific context, what data and methods are appropriate, readily available or can be collected within time and budget constraints. A logic model /conceptual system map of anticipated implications across sectors and scales should help identify optimal study design, analytical techniques and measures. These models should be updated when synthesising findings across diverse methods and integrating findings across subpopulations using similar methods.

**Supplementary Information:**

The online version contains supplementary material available at 10.1186/s12889-021-11984-2.

## Background

There is ample evidence showing that consumption of sweetened beverages is strongly and positively associated with the prevalence of non-communicable diseases (NCDs), including obesity, type 2 diabetes, cardiovascular diseases [1–3] and certain cancers [4, 5], as well as all-cause mortality [6]. Consumption and outcomes are socio-economically patterned and lead to health inequalities [7]. Consumption of these beverages is higher than recommended [8] and increasing, particularly in low- and middle-income countries [7]. In higher-income countries, where there have been initial reductions in sugar-sweetened beverage consumption, these reductions have plateaued and the beverage industry is failing to meet its own pledges [9]. Effective regulations will be needed to discourage sweetened beverage consumption so as to contribute towards curtailing the pandemic of associated chronic NCDs and obesity. If not tackled, these diseases will continue to have severe consequences for healthcare systems and economic development. Poorer individuals will generally bear the greatest burden of associated morbidity and mortality [10, 11]. In recognition of these challenges, there is growing global momentum to use pricing policies, such as sweetened beverage taxes, as a key intervention to help address the global NCD pandemic, make progress towards achieving the Sustainable Development Goals [12] and reduce inequities [10, 13–16]. These efforts have been backed by an abundance of academic studies, systematic reviews and meta-analyses [17–21].

As these sweetened beverage taxes are passed and implemented [22], it is important to assess whether and in what ways the various forms of these policies contribute towards changing consumer demand and industry supply of beverages, their intended and unanticipated health, economic, equity and broader wellbeing impacts, and what improvements may be made to strengthen and maximise the their success. This requires rigorously planned and executed evaluations.

In this article, rather than provide a review of the findings of existing evaluations [23, 24], we ask: what are the lessons we can learn from sweetened beverage tax evaluations that can help improve future evaluations and thus support NCD and obesity prevention efforts? To this end, we offer an overview of progress in the field and an analysis of key theoretical, design and methodological considerations, recognizing that it is unlikely that any single evaluation of sweetened beverage taxes will be fully comprehensive given data, resource and time constraints. As such, we do not seek to elevate one evaluation approach over others or rank them, rather we hope to describe the various trade-offs and constraints under which such real-world evaluations function under and thus the issues potential evaluators should consider given their particular context and circumstances. We limit our narrative review to existing evaluations where: there has been a clear declaration of interests which demonstrates that the research team is fully independent and has no conflicts of interest with regard to the findings; and where a scientific or advisory committee have been formed to provide independent oversight of the evaluation [25].

### Understanding sweetened beverage tax policies

Understanding the characteristics and nuances of sweetened beverage tax policy designs, including their genesis, development, political context, and stakeholder involvement in influencing their goals and design, form the backbone for developing an evaluation approach. Table 1 provides selected examples from over 45 jurisdictions that have implemented such taxes as of August 2021 [22], illustrating the different types of sweetened beverage tax structures, regulatory designs and their explicit and implicit goals to illustrate how and why these are important.

**Table 1:** Examples of sweetened beverage tax structures, designs and their goals

Tax design	Levied on/ collected from [goes to] [22, 26]	Example where implemented						
		Location	Dates [22, 26]	Taxable categories definitions [22, 26]	Exempted products/ Categories [22, 26]	Tax rate/level [22, 26]	Stated purpose	Framing
Ad valorem	Excise on distributors, importers & manufacturers [general budget]	Barbados [27] [national]	Announced: June 2015 Implemented: 1 September 2015	Carbonated soft drinks, juice drinks, sports drinks, fruit juices that contain added high calorie sweeteners based on Harmonized System (HS) tariff codes	• 100% juices • Powders and concentrates • Sugar sweetened dairy/milks	10% of value/price that manufacturer/distributors charge retailers.	Address the high burden of non-communicable diseases in Barbados [27]	Revenue generation Reduce consumption of taxed beverages [27]
Ad valorem	Goods and Services Tax (GST) collected at point of purchase [general budget]	India [28] [national]	Implemented: 1 July 2017	Aerated drinks and lemonades based on HS tariff code	• 100% juices • Powders and concentrates • Sugar sweetened dairy/milks • Other non-aerated drinks with added sugar	Aerated drinks and lemonades (40%: from 12% GST on all processed packaged foods/beverages + 28% GST on sin goods)	Simplify tax structure of prior Central VAT + State VAT, and eliminate cascading taxes [28]	Improve tax collection and revenue generation [28]
Specific – Volume	Excise on distributors, importers & manufacturers [general budget]	Mexico [29] [national]	Implemented: 1 Jan 2014	All non-alcoholic beverages with added sugar including reconstituted powdered sugar-sweetened drinks and flavoured/sweetened dairy products that are not milks	• Milk products (milk is the primary/first ingredient) • 100% fruit and/or vegetable juice	1 Mexican Peso per ready-to-drink litre Indexed to inflation once cumulative inflation hits 10%	Address high prevalence of diabetes and cardiovascular diseases in Mexico [29]	Reduce consumption of taxed beverages [29]
Specific – Volume	Excise on distributors, importers & manufacturers [Office of education and general budget]	Philadelphia County/City in Pennsylvania, United States [30] [City Council vote]	Enacted: 16 June 2016 Implemented: 1 Jan 2017	• Bottled beverages • Syrups/concentrates for commercial sale • Fruit/vegetable drinks with added sugar • Mixers • Coffee syrups distributed to coffee shops • Beverages containing sweeteners that are only non-caloric (“diet drinks”)	• Milk products (milk is the primary/first ingredient) • 100% fruit and/or vegetable juice • Syrups, concentrates, and powders sold to consumers • Natural or common sweeteners that are not already in beverages	US $0.015 per ounce on retail sale on ready-to-drink volumes of taxable beverages Not indexed to inflation	None Earmark not in ordinance but in mayor’s budget [26].	Revenue generation to support new or expanded programs including Pre-K access and Rebuild (for park, community centre, and library repairs) [26].
Specific – Volume tax based on sugar concentr-ation threshold	Excise on distributors, importers & manufacturers (exempt producers with < 1 million litres/year) [general budget]	United Kingdom [31] [national]	Announced: March 2016 Public Consultation: Aug 2016 Implemented: 6 April 2018	All packaged beverages that contain sugar added during production of at least 5 g of sugar per 100 ml in ready to drink form	• Drinks with ≥75% milk • Milk replacement drinks (e.g. plant based ‘milks’) • Alcohol replacement drinks (with alcohol by volume < 0.5%) • 100% fruit/veg juices • Liquid drink flavouring added to food/drinks • Powders mixed into liquids and served in open container	£0.18 per litre for drinks containing at least 5 g of sugar per 100 ml £0.24 per litre for drinks with more than 8 g per 100 ml.	Reduce childhood obesity by removing added sugar from soft drinks [31]. Encourage soft drink producers and importers to [31]: • reformulate to cut sugar content • reduce portion sizes of added sugar drinks • import reformulated drinks with less sugar	Reformulations Reduce sugar consumption from beverages Fund children’s health initiatives (e.g., school sports and healthy school breakfast clubs) [31]
Specific – Sugar content based	Excise on distributors, importers & manufacturers (exempt producers with < 50 K liters/yr) [general budget]	South Africa [32] [national]	Policy paper for public consultation: July 2016 Draft legislation: Feb 2017 Enacted: Dec 2017 Implemented: 1 April 2018	Based on Harmonised System (HS) tariff codes • Syrups and concentrates • Cocoa powder and milk extracts • Non-alcoholic waters, mineral, aerated or juices, with sugar or flavouring • Non-alcoholic beer	• Milk products (milk is the primary/first ingredient) • 100% fruit and/or vegetable juice	2.1 SA cents per gram of sugar in excess of 4 g/100 ml based on ready-to-drink (reconstituted) form Products in taxable HS category with no sugar information will be taxed based on default of 20 g of sugar/100 ml in reconstituted form Indexed to inflation	Address high prevalence of diabetes, obesity and cardiovascular diseases [32, 33]	Reduce sugar consumption from beverages [32]
Specific – Sugar type based	Excise on distributors, importers & manufacturers [general budget]	Philippines [34] [national]	Enacted: 19 Dec 2017 Implemented: 1 Jan 2018	Sweetened pre-packaged: • Sweetened juice drinks • Sweetened tea • All carbonated beverages • Flavoured water • Energy and sports drinks • Powdered drinks not classified as milk, juice, tea or coffee • Cereal /grain beverages • Other non-alcoholic beverages with added sugar	• All milk products, whether powdered or in liquid form, sweetened or not • 3-in-1 coffee packs • 100% fruit and vegetable juices • beverages sweetened with stevia or coconut sugar.	Drinks with caloric and non-caloric sweeteners will be taxed 6 Ph Peso per litre. Drinks with high-fructose corn syrup taxed at 12 Ph Peso per litre. Not indexed to inflation	Generate revenue and fight obesity and diabetes and poor dental health Part of larger Tax Reform for Acceleration and Inclusion (TRAIN) Law [34]	Health measure to addressed poor oral health which results in poor school attendance and poor nutrition Improve tax collection and revenue generation [34]

Tax structure and design critically informs evaluation design and reflects the primary objectives of the tax. Who the tax is levied on (e.g., large vs small vs all manufacturers/distributors, large, vs small vs all retailers, or consumers) will determine the research questions and the types of data needed. Additionally, whether the tax is *ad valorem* (percentage-based) or specific (unit-based), and what the chosen tax base is (e.g., value-added, pre-tax price, volume, sugar-content) determine the measures to track. The tax design also matters in terms of how it (dis)incentivises changes. For example, is the tax a flat rate (e.g., 10% as in Barbados or 1 cent/ounce, as in Berkeley California), a linear rate (e.g., as in South Africa) or multi-tiered (e.g., as in the UK)? For flat-rate taxes, monitoring changes in prices, sales and/or purchases might suffice, but researchers should consider, theorise and monitor alternative ways that industry may respond (e.g., shrinking package sizes while maintaining or increasing price, strategic cost shifting across beverage types and size offerings, marketing and promotions). For taxes based on sugar content, it will be important also to monitor changes in sugar content of products (reformulation), manufacturers’ portfolios (e.g., product innovation, changes in package sizes) by sugar concentration and marketing of their products including labelling and claims, and promotions based on price or volume.

Also, does the tax apply to 100% juices, milk-based drinks, alcoholic drinks (alcopops) and artificially sweetened beverages? For sugar-concentration based taxes, do these apply to products with intrinsic sugar (e.g., dairy, fruit juices)? Data on the distribution of sales by type of beverages can help in designing a tax that covers all potential substitute drinks. It also provides valuable information on the potential untaxed beverages (e.g., water, plain milks) that could be substitutes and will need monitoring. The definition of products included versus excluded from the tax also then needs to be matched with evaluation data with sufficient detail to classify products appropriately since misclassification of taxed products as non-taxed (or vice-versa) will bias results.

The geographical jurisdiction of the tax policy may impact on the extent to which cross-border shopping might exist. Generally, the smaller the political or physical geographical scope, the more attention should be paid to monitoring cross-border behaviours of consumers, distributors and retailers.

The framing around the purpose of the tax and how the tax policy is made known to the public by legislators and advocates also has implications for the evaluation design. On the first point, there may be multiple objectives of sweetened beverage taxes, such as lowering sweetened beverage consumption, improving health, generating revenue for general uses or specific uses like health promotion and/or early childhood education. The political context and public opinion or support likely drives this framing, and the name of the tax policy can help convey the chosen framing. For example, in the UK, the term “Soft Drinks Industry Levy” (SDIL) conveys that the levy is placed on industry and that the primary purpose is to encourage supply-side changes (rather than to change the public’s behaviour). This means that the evaluation design should likewise prioritize careful monitoring of industry responses [31, 35]. On the second point, how the tax is made known has implications particularly around the salience of the tax policy and signalling effects [36]. In the case of sweetened beverage taxes, higher salience likely promotes both consumer demand and supply changes.

Finally, from a policy perspective, while a sweetened beverage tax is typically one of the first food policies to be considered for addressing and preventing NCDs and obesity in any jurisdiction, it is often not the only policy considered. The timing and sequence of policies should be considered carefully as this will have implications for evaluation. For example, when policies are implemented together or in close succession, then it becomes challenging to measure the impact of each policy, so it would be important to consider what analytical approach makes sense for multi-intervention type evaluations to better distinguish the effects of each.

### Evaluation stages and considerations

Because sweetened beverage tax policy development, legislation and implementation are events in complex adaptive systems, it is beneficial to take a logical, sequential and systemic approach to considering evaluation. This includes assessing the evaluability of the policy; theorizing the policy’s impacts across sectors and scales (micro vs macro), as well as theorising both intended and unintended consequences from a public health perspective (e.g., product innovation or additional marketing that avoids the tax, but has the potential to worsen or harm health); identifying the optimal study design, analytical techniques, data and measures; and bringing the various components of work back together via interpretation, synthesis and integration.

**Fig. 1:**
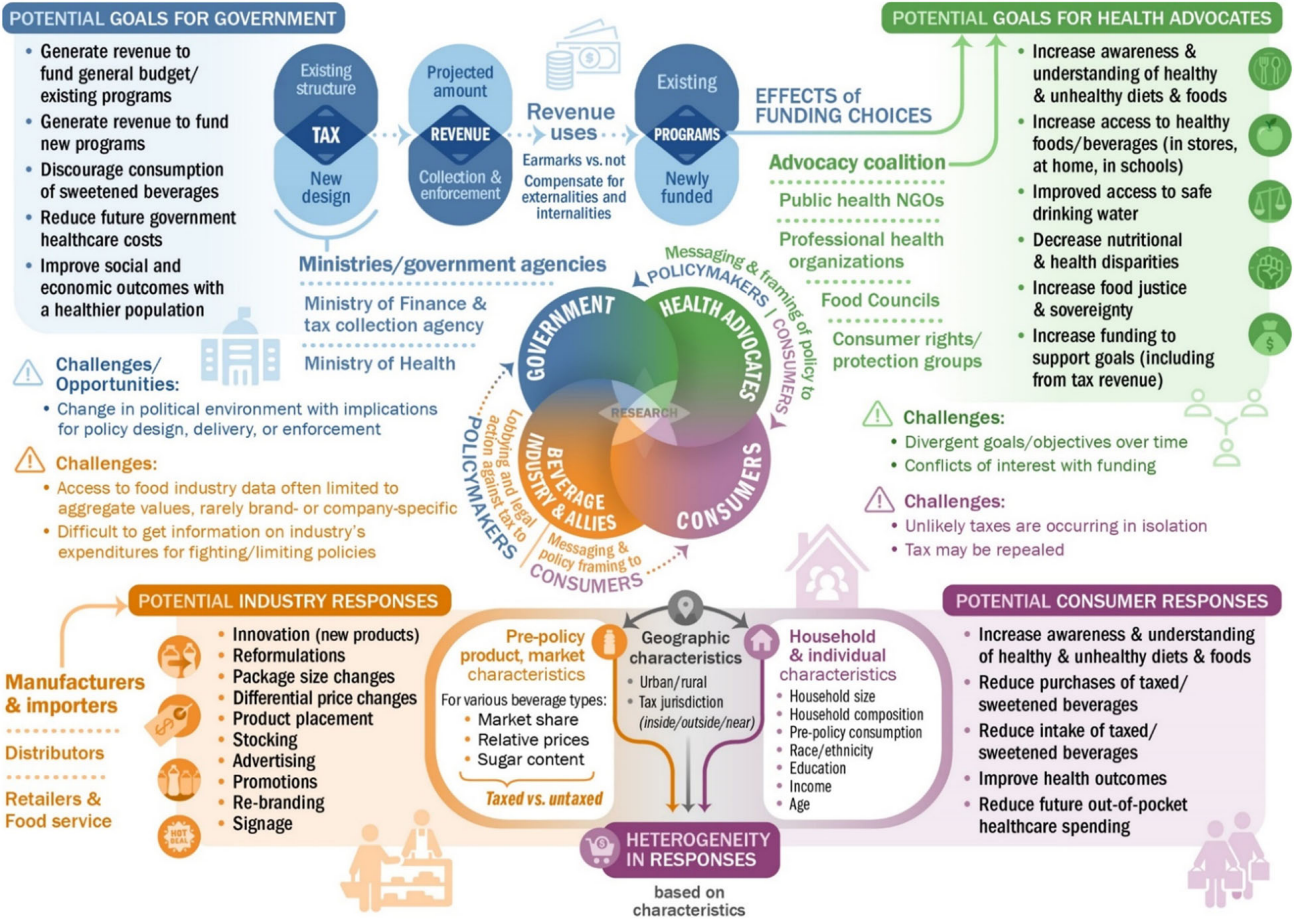
Stakeholders for sweetened beverage tax evaluations, potential outcomes and responses. Note: Graphics used in this Figure were designed and created using a licensed copy of Adobe Illustrator 2020 and include adapted icon graphics from Flaticon.com

Figure 1 illustrates graphically the key stakeholders that need to be engaged in evaluation efforts, the challenges they present to researchers and evaluations and, for each constituency, the potential for impacts of a sweetened beverage tax and related potential data sources. We refer to this further in the sections below. Our view is that engaging stakeholders in all aspects of a study, from conception, through design to execution, interpretation and knowledge exchange is critically important to ensuring the research is grounded in reality.

#### Assessing Evaluability

Before researchers, policymakers or funders move on evaluating policies or interventions, it is beneficial to undertake an evaluability assessment to determine the extent to which an evaluation is feasible and build consensus among stakeholders (central section of Fig. 1) about the need, value and purposes of evaluative research [37]. Evaluability assessment is critical for the process of prioritising research questions together with stakeholders. In undertaking evaluability assessment, it helps to consider the following five questions, proposed by Ogilvie et al. [38]. Resolving these uncertainties will help to ensure the conduct of a viable and worthwhile evaluation:

1. Where is this policy/intervention situated within an overall (NCD prevention) program/strategy?
2. How will an evaluation study of this policy/intervention affect policy decisions?
3. What are the plausible sizes and distribution of the policy’s/intervention’s hypothesized impacts?
4. How will the findings of an evaluation study add value to the existing scientific evidence?
5. Is it practical to evaluate the policy/intervention in the time available?

#### Developing a conceptual understanding of the tax policy

Understanding the dynamic systems that the sweetened beverage tax is likely to affect and hypothesising potential impacts across sectors and populations is an important first step. This can be supported by conceptual system mapping, developing a programme theory or logic model, or another form of causal diagram (e.g., a directed acyclic graph, DAG) [39–41]. In undertaking this process, it helps to engage with complexity theory and systems thinking, to theorise fully the range of possible reactions and counteractions that the policy will stimulate, especially from industry [42].

Involving multiple stakeholders (Fig. 1) in such activities via deliberative processes helps to ground conceptual thinking in the present context and build trust in the evaluative process. This can take the form of key informant interviews, focus groups, content and media analyses of public and trade press around the framing of the issues, review of legal documents, potential data or leaked documents on industry’s lobbying, legal or other subversive strategies or actions [43], and consensus building processes (e.g., using community-based participatory research methods, Delphi studies or group model building activities). One example is a conceptual mapping process and Delphi study conducted in the UK to support evaluation of the SDIL [44]. Another example is a qualitative assessment of the tax passed, implemented and then repealed in Cook County Illinois [45].

Key informants might include technical and political staff at revenue agencies and ministries (e.g., finance, commerce/trade and industry, health) who have interests or involvement in the tax, those in food industry (manufacturers, retailers, food service sector), public health professionals (e.g., professional organizations, local food councils) and advocates (e.g., public health advocacy coalitions, consumer rights groups), and of course the public (especially those most affected by NCDs). Among the public, it will be instructive to consider various subsamples based on socio-demographic characteristics, political persuasion or other characteristics that might influence awareness of the proposed or implemented tax, understanding of tax (e.g., rate, scope), support of the tax or anticipated response to the tax [46]. Depending on the framing of the tax policy (central portion of Fig. 1), other key proponents might include those who could benefit from the tax revenue (e.g., school board members, teachers, parents), while other key opponents might include individuals who may perceive becoming disadvantaged (e.g., distributor or retail employees, advertising agencies, sugar producers).

The messaging around the tax among both those for and against the policy requires careful analysis to understand the underlying concerns and potential reactive strategies among stakeholders. Very often contextual factors such as the degree of trust in the government, agencies and politicians, the degree of coherence and coordination among those on each side of the issue, and concerns around food sovereignty, equity and policy efficiency deserve careful attention [47, 48]. Moreover, such analyses may uncover new perspectives or angles that researchers may not have considered. How a policy is framed by legislators and others can impact the public’s responses to a tax; for example, a policy framed to address key public concerns (e.g. use of revenue or equity) could better garner public support and incentivize behavioural change [49], compared to one viewed as increasing burden to the public due to mismanagement of resources [45]. Given the likely disparate viewpoints from various stakeholders, researchers should also develop a communications plan in the early stages of designing an evaluation to minimize distraction.

#### Choosing the optimal study design and analytical techniques

##### Process evaluation

Research to assess the adoption, extent and fidelity of implementation of a tax should be considered part of an evaluation effort, especially when the tax structure is complex. Process evaluation allows us to complement outcome and impact evaluation by assessing why a tax policy has achieved its intended impacts or not, as well as to assess for whom the policy is beneficial or otherwise, and under what circumstances [50]. This includes assessing whether there is clear language and guidance on who the tax should be collected from and how frequently, which products are taxed, how the tax is calculated, what the penalties are, the timeline for implementation, how tax revenue use is being determined and which government entities are charged with conducting and overseeing and inform/communicate to the affected industries and the public about the tax collection, reporting, enforcement and revenue use (upper sections of Fig. 1 – in blue and green). Moreover, the role and engagement of other stakeholders like the beverage industry and its allies (e.g., trade associations), distributors and retailers, and public health advocates in each jurisdiction and how they interacted with the media to frame their positions to the public and to policymakers also matters (middle section of Fig. 1). Implementation science approaches such as Consolidated Framework for Implementation Research are useful for uncovering what nuanced on-the-ground factors may matter, such as what has been done in understanding the enablers of success (and room for improvement) in Berkeley California [51] and causes of failure in Cook Country Illinois [45].

##### Natural experimental evaluation methods

Since sweetened beverage taxes are implemented at some level of administrative jurisdiction, natural experimental methods are likely the most appropriate approaches to use for evaluation; and how they are applied will be a function of available data [52]. These can be used for assessing both micro-level outcomes like individual intake or household purchases [23], and macro-level outcomes like un/employment or revenue generation and use [53]. There is a substantial literature that describes these methods and how the various approaches available can help strengthen causal inferences [54–57]. Table 2 lays out some of the key analytical designs (difference-in-differences, interrupted time series, regression discontinuity), statistical approaches (propensity scoring, correlated random effects), and examples where they have been used. Robustness checks, sensitivity analyses and, if possible, analyses using different data sets and considering different time frames are recommended to ensure that the results are stable and will not vary wildly when minor changes are made to how definitions are operationalized and to ensure that results are not being driven by outliers or choice of comparison population or sites.

**Table 2:** Examples of natural experiment methods used for evaluating sweetened beverage taxes

Study design (with statistical method)	Advantages	Challenges	Example of evaluations
Difference in difference (using a control group)	Reduces biases associated with time-varying factors related to the outcome of interest.	Requires that prior trends of the outcome are similar between treatment and control groups. Difficult to test if no prior data available.	Philadelphia (USA): the evaluation of the tax was based on a difference in difference analysis to estimate changes in sales, using Baltimore as the comparison city [30].
Difference in difference (with propensity score matching)	In absence of an experimental design PSM balances control and treatment comparison groups on basic characteristics using baseline data.	Unable to adjust for unobservable time variant variables.	Philadelphia (USA): Created propensity score weights as inclusion in difference-in-difference models to account for differences in the composition of the four comparison groups and changes in their composition over time [58].
Interrupted time series (ITS)	Creates a counterfactual based on pretax trends. Can be adapted to panel and cross-sectional data.	No control group to adjust for all potential exposures to other policies or factors associated with the outcome of interest.	Mexico: Adapted ITS to a panel of urban households to estimate changes in household beverage purchases, using a fixed effects regression and adding household and contextual variables [29]. UK: Controlled ITS to look at sugar content, prices and beverage product availability from 2 years pre-announcement to 1 year post-implementation [59]; Domestic turnover of UK soft drinks manufacturers pre-post announcement and implementation of the SDIL [60].
ITS with synthetic controls	Creates a synthetic control based on a pool of potential comparison groups.	Requires countries with same data sets for the outcome and variables associated with the outcome prior to the intervention to create the synthetic control. Requires the magnitude and trends in the pretax period are not statistically different between treatment and synthetic control	Mexico: Uses Mexico’s Consumer Price Index price data collected from urban retail outlets across 46 cities to construct a synthetic control product whose pre-tax price most closely tracks that of the treatment product (‘donor’ products comprised of all untaxed non-durables that are neither potential substitutes for taxed drinks nor subject to the concurrent junk food tax) [61].
ITS with correlated random effects	Adjusts for unobserved heterogeneity at the household level. Can be combined with ITS approaches to adjust for pre-intervention trends.	No control group to adjust for all potential exposures to other policies or factors associated with the outcome of interest.	Chile: estimated changes in beverage prices and purchases associated with a tax policy modification in a panel of urban households adapting a ITS model with a correlated random effects model [62].
Regression Discontinuity (RD)	Uses cutoff score on a pre-policy measure to determine allocation of treatment vs control and thus removes potential selection biases and increase internal validity of results.	Requires cutoff to be exogenous (not linked to outcomes). Results more relevant for observations around cutoff (external validity can be difficult to establish)	Denmark: Uses a regression discontinuity (RD) approach to assess the pass-through of the tax changes and a within-household pre-post design to estimate changes in purchases of soft drinks [63].

##### Simulation methods

Simulation models serve as important adjuncts to empirical evaluations, particularly for more distal or longer-term outcomes such as lowering NCD prevalence and mortality, healthcare cost savings, how these may vary across lower vs higher income populations [64], as well as macro-economic factors (e.g., employment or revenue use) [65]. Simulation modelling can be helpfully combined with empirical evaluation and is especially powerful when measured intermediate outcomes (e.g., changes in purchasing or consumption) are used to parameterise models. Simulation models also allow for comparison of various policy options. In fact, much of the earlier evidence used to make the case for sweetened beverage taxes was based on using demand system models [66], life tables [67], microsimulations [68, 69], extended cost-effectiveness analyses [70], or input-output models [71] under various scenarios.

Some industrial organization (IO) approaches that use parametric or semi-parametric models to simulate beverage demand and supply jointly are also useful to consider [72]. These models consider how firms may respond to taxes via price changes given their market share across beverage types and also how the public would thus change their purchases or intake to reach new equilibriums [73]. With the implementation of sweetened beverage taxes in some jurisdictions, it is now possible to validate these models and, if they perform well, these models can then be used for simulating higher rates or different policy designs. Table 3 lays out some of the key simulation-based methods and examples of how and where they have been applied.

**Table 3:** Examples of simulation-based methods used for distal outcomes or comparing sweetened beverage tax designs

Simulation-based methods	Advantages	Challenges	Example of application
Demand systems models such as Almost Ideal Demand System (AIDS); Quadratic Almost Ideal Demand System (QUAIDS), Rotterdam model, Exact Affine Stone Index model (EASI)	Accounts for simultaneous substitutions and complementarity across beverage & food categories to obtain own- and cross-price elasticities of demand as well as income or expenditure elasticities If sample large enough, able to disaggregate by income or other characteristic of interest to get subpopulation specific elasticities.	Elasticity estimates may be sensitive to how model used, beverage/food categories included in the system and sample sizes. Beverage/food categories that are not often purchased require more complex econometric models. Demand models are usually static and does not account for habit formation.	Connecticut & Massachusetts, USA: Uses QUAIDS to estimate SSB demand and tax among food assistance participants [74]. Chile: Uses LAIDS to derive beverage and food elasticities to infer what a tax might mean in terms of changing consumption [75].
Demand- and supply-estimations for differentiated product markets using the Berry, Levinsohn and Pakes (BLP) model	Able to account for beverage market structure and shares of firms (manufacturers and/or retailers) and household socio-demographic characteristics to assess how much of the tax is passed onto consumers (strategic pricing) and hence what the resultant consumption changes accounting for substitutions, and new market share distribution might be. If a tax is in place, possible to validate the structural demand and supply models.	Requires detailed data on product attributes (e.g., brand, tax status) purchased by households. Determination of inside- vs outside-option may limit interpretation as the model assumes that the price of the outside option is unchanged. Only provides estimates of short-term supply-side response to a tax as other strategies like changing portfolio mix and reformulations may follow. Demand models are usually static and does not account for habit formation.	France: Comparing firms’ strategic price responses to an ad valorem vs excise taxes on sweetened beverages [73]. Mexico: Comparing changes in volume of and sugar from taxed vs untaxed beverages purchased as well as tax revenues generated under SSB taxes based on sugar-density vs volume [76].
Population-based microsimulation models (PSM) of which extended cost-effectiveness analyses (ECEA) are a subset	Uses existing distribution of population characteristics collated from various data sources to construct a hypothetical population. Various policies or interventions and empirically informed effect sizes between dietary intake changes and health outcomes are applied as parameters to compare how outcomes would vary across these policies or interventions vis-à-vis the status-quo.	Assumes that diseases are independent of each other. Frequently due to data limitations, only key diet-disease relationships are included in models Unable to account for industry responses such as reformulations or changes in marketing. Need to define time horizon given population cohort and assume discount rates. Validation of assumption and methods needed but often difficult.	USA: Applies the CHOICES model to estimate cost-effectiveness of a 1 cent/ounce tax on SSBs [70]. Australia: Multi-state lifetable model of a hypothetical 20% SSB tax on the monetized productivity of adults 20y or older [77]. South Africa: Estimates changes in Type 2 Diabetes-related deaths for different income groups and the resultant burden to individual and public payers due to a 20% SSB tax [78].
Computable general equilibrium (CGE) models of which input-output models or social accounting matrix are a part of.	Able to assess macroeconomic/economy-wide implications (employment, sector-specific productivity, trade, gross domestic product) using representative agents (consumers, producers, government) and accounts for import-exports for country	Assumes that demand elasticities are fixed and independent of policy Requires additional parameters from demand systems model estimates, market share changes, PSM and cost-effectiveness estimates and thus only possible later in the lifecycle of the evaluation.	Guatemala: Considers the whole value chain, from the production of sugar to the different productive sectors that use sugar and the final consumer to evaluate the overall effects various SSB tax policies [79].

#### Identifying the appropriate outcome measures, data and timing

While determining the optimal analytical methods, researchers need also simultaneously to consider potential outcome measures and understand what data are available. Figure 1 illustrates what the potential goals of government and health advocates and potential responses by the beverage industry and consumers might be. For example, the revenue generated from the tax (a potential goal by government) could be used in ways that create multiplier effects for the economy or help narrow existing health disparities (a potential goal for health advocates), such as been done in Seattle in response to the COVID-19 pandemic’s impact on low-income families [80]. The lower section of Fig. 1 (orange and purple) illustrates the potential responses by the beverage industry and consumers, which may be more immediate or lagged. This means that some components of complex interventions could or should be evaluated later in the timeline of the policy (and thus other contextual factors that may have evolved over that period will also need to be considered). Resultant micro or macro as well as time period specific outcomes of interest in evaluations of sweetened beverage taxes are show in Table 4, and the data sources to assess these outcomes, as well as their strengths and limitations, are further elaborated in the Supplementary file.

**Table 4:** Outcomes or measures of interest by stakeholder

Stakeholder	Consumers		Beverage industry and allies	Government	Health Advocates
***Time periods***	*Pre-tax and < 5 years post-tax implementation*	*Pre-tax and > 5 years post-tax implementation*	*Pre-tax and post-tax implementation*	*Post-tax implementation*	*Pre- and post-tax implementation*
**Micro**	• Awareness, understanding, and support of tax • Knowledge of health implications unhealthy beverage consumption • Purchases or intake of taxed and untaxed beverages in terms of volume, sugar and calories • Total sugar in diet and compensatory consumption of other foods or beverages • Select biomarkers (e.g., HbA1c)	Continue earlier measures to assess sustainability of consumer changes	• Marketing practices (mix of product, price, placement and promotion). o Price changes relative to liable tax (degree of pass-through) o Sugar content of beverages o Product offerings across beverage types, formats, markets, regions o Types of signage or messaging on receipts about tax, by store type • Store revenue overall and for beverages • Costs for research & development, new packaging	Implementation, administration, collection and enforcement of tax: • Guidance to stakeholders • Mechanisms for collection and enforcement • Communication of goal of tax to the public • Costs of above	• Programs supported by tax revenue allocation and impacts on community • Composition and coherence of advocacy coalition
**Macro**	• Affordability of taxed vs untaxed beverages • Modelled simulations of longer-term health and healthcare cost outcomes	Empirical measures of incidence & prevalence of: • Type 2 diabetes • Dental caries	• Employment by/across industries • Market share of key beverage types • Market value (stock prices) of companies • Expenditures on lobbying and legal action to fight or repeal tax	• Tax revenue collected • Tax revenue allocated over time and by purpose • Political acceptability of wider policy measures and government’s role	• Expenditures on campaigning and legal action to support tax • Perceptions and attitudes on the role of advocacy and civil society

#### Practical challenges of complex evaluations

There are of course real and practical challenges in evaluating sweetened beverage tax policies, including:

- Data availability – detailed data with valid measures for outcomes, sufficient sample sizes and time periods of interest may not exist.
- Time pressures – primary data collection may need to occur extremely quickly if there is a short time lag between policy passage and implementation, which may prevent researchers from collecting baseline (pre-implementation) measures of interest. This is more so the case if there are difficulties or delays in obtaining funding for primary data collection. Moreover, these tax policies are often heavily scrutinized and there is pressure to get results out quickly. Thus, it is wise to conduct routine quick assessments of all data possibilities and prioritise data that is available. This also means that researchers and policymakers should be in constant dialogue to ensure timelines are realistic.
- Costs (data, personnel) – primary data collection requires both financial and human resources and tends to have finite windows of opportunity for collection, so obtaining funding to cover these costs quickly can be difficult. Funders interested in these issues need to establish mechanisms with quick turnarounds for supporting evaluations. Commercial data may also be expensive to obtain and may require more personnel time to familiarise and analyse (see Supplementary file).
- Political acceptability – sweetened beverage taxes may be viewed unfavourably by governments with a more libertarian ideology, and this view is often promoted and supported by industry through media framing [81, 82]. In such circumstances, arguments are often made about the problems of the ‘nanny state’, job losses and regressivity of a tax. Besides generating evidence to address these concerns (e.g., via data analyses, evidence synthesis and modelling), researchers can readily counter such arguments in discussing their findings, thus presenting an alternative framing based on the science [83].
- Obtaining conflict-free funding – it is important that objective and independent evaluations of sweetened beverages taxes take place, without interference from those with vested interests [82, 84–87]. Gaining support for such evaluations in the relevant setting may be difficult, but support may be available from government sources, private philanthropies or foundations, global research funds or a mixture of these sources. Care should be taken to consider all potential forms of interests to establish ways to mitigate or at the very least, disclose them [86, 88, 89].
- Media attacks – vested interests (e.g., political think-tanks, industry foundations) and elements of the public media are often aligned with government or opposition views. Either side may attempt to discredit evaluations of sweetened beverage taxes to which they are ideologically opposed through media articles [90]. As indicated above, such attacks can be countered, but sometimes are meant to distract from the work at hand, and may be best ignored by researchers and/or countered by other stakeholders (e.g., advocacy).
- Communication of evaluation findings beyond academia – the complex methodologies of rigorous research can create confusion if not communicated well. However, while access to policymakers and other stakeholders can be difficult, it is valuable for researchers to communicate their findings to a wide audience beyond academia, using multiple channels to maximise impact of their research.

Given these challenges, researchers, policymakers and funders should be mindful and realistic in the scope of evaluations, keeping the following questions in mind to guide decisions:

- How unique is the tax policy design or context?
- What research capacity/know-how exists, given the timeframe, to execute the work?
- What are the existing knowledge gaps and critical uncertainties that your research might uniquely be able to address?
- How can your evaluation contribute more widely to generalizable causal inference regarding sweetened beverage taxes?
- What is the anticipated time-horizon by which any meaningful changes in outcomes should be expected? Will there be resources to do conduct a study over this time-horizon?

#### Making sense of the findings

Once new evidence has been generated on the implications of the sweetened beverage tax for outcomes of interest, it is then useful to revisit the prior programme theory, conceptual map or logic model. This allows evaluators to:

- Interpret the findings across multiple outcome measures in relation to a given context, theory of change or expected results (e.g., the Obesity Prevention Evidence Assessment Framework [37])
- Explain unexpected results
- Synthesize findings from multiple methods (including using formal methods, such as triangulation protocol [91], pattern matching [92] or process tracing [93])
- Integrate findings from multiple similar analyses using integration methods (e.g., meta-analyses of multiple interrupted time-series analyses [23, 53, 94])

Returning to stakeholders to review the findings and to make sense of any counterintuitive findings by identifying nuances that the data might not have captured will be important as well as informative for future evaluation efforts [95].

All stakeholders, including researchers and funders, should be fully cognisant of study limitations given that no single source of data, method or evaluation will be perfect or necessarily produce generalizable findings. Even consistent findings across multiple methods for a said location or policy do not necessarily mean that that same policy implemented elsewhere will produce similar results. There are lessons learned from elsewhere to inform policy development, but local context also matters and needs to be taken into account. This is particularly true when taking a systems approach to understand how stakeholders interact or respond [95, 96].

## Conclusions

Careful evaluation of public health policies can generate evidence to support the refinement of existing policies and inform the development of new policies elsewhere. The guidance provided here builds on lessons learned to date from a range of evaluations of sweetened beverage tax policies. We anticipate that it will help in planning future evaluations of sweetened beverage taxes and, in addition, be applicable to the evaluation of other food policies, like food labelling or marketing regulations. We hope that this article will help researchers and policymakers consider how to prioritise evaluation questions and choose appropriate study designs and methods, given potentially limited data, resources and time, and hence the practical trade-offs that they may need to decide among within each context. We recommend adopting a systems perspective, incorporating insights from multiple disciplines and stakeholders, developing a communications plan, and being creative in identifying a mixture of data sources and applying diverse methods informed by systems thinking, involving a range of relevant stakeholders at each step of the evaluation process.

## Supplementary Information

**Additional file 1.**

## Data Availability

Data sharing is not applicable to this article as no datasets were generated or analysed during the current study.

## Abbreviations

AIDS: Almost Ideal Demand System
GST: Goods and Services Tax
HS: Harmonised System
ITS: Interrupted Time Series
NCD: Non-Communicable Diseases
PSM: Population-based Simulation Models
QUAIDS: Quadratic Almost Ideal Demand System
RD: Regression Discontinuity
SDIL: United Kingdom’s Soft Drink Industry Levy
SSB: Sugar Sweetened Beverage
VAT: Value- Added Tax
