# Supplementary material for: How should we evaluate sweetened beverage tax policies? A review of worldwide experience
Source: BMC Public Health. 2021 Oct 26;21:1941. doi: 10.1186/s12889-021-11984-2 (PMC8546197; doi:10.1186/s12889-021-11984-2)
Supplement: Supplementary file 1 — Additional file 1. [file 12889_2021_11984_MOESM1_ESM.docx]

**Supplementary File**

**How should we evaluate sweetened beverage tax policies? A review of worldwide experience**

Shu Wen Ng ^1*^, M Arantxa Colchero ^2^, Martin White ^3^

^1^ Department of Nutrition and Carolina Population Center, University of North Carolina at Chapel Hill, CB #8120, 137 East Franklin Street, Chapel Hill, NC 27516; shuwen@unc.edu

^2^ Center for Health Systems Research, Instituto Nacional de Salud Pública, Universidad No 655 Colonia Santa María Ahuacatitlán, Cerrada Los Pinos y Caminera. CP 62100, Cuernavaca, Morelos, México.

^3^ Centre for Diet and Activity Research, MRC Epidemiology Unit, University of Cambridge, Cambridge, United Kingdom

1. **Primary data collection**

Primary data collection should include qualitative as well as quantitative data. Well conducted early qualitative work that informs the development of a logic model or conceptual map can also serve as the baseline for qualitative analyses of emerging discourse on the tax among key stakeholders, including on acceptability. Follow-up qualitative semi-structured interviews and focus groups with key stakeholders after tax implementation can determine if there are new themes or issues that have arisen compared to earlier qualitative work. This will provide insights on whether the logic model needs updating and what additional (primary or secondary sourced) outcome measures should be included in the quantitative work.

Primary data collection may be challenging if the tax passage and implementation window is short, so whenever possible, planning in anticipation is highly recommended. Researchers need to decide if there are specific stakeholders or subpopulations (e.g., rural vs urban; children, adults or all ages; low income; high consumers) or segments of beverage demand/supply (e.g., away from home, food service, small stores) they will focus on either due to gaps in the literature and/or due to resources constraints. The mode of population-based data collection also matters as there are implications for representativeness. Options include telephone-based, web-based, and in-person samples recruited from certain locations. We encourage a thoughtful approach to sampling frames and caution against the use of convenience samples. This is because the representativeness of convenience samples even after applying post-sample weights may still be problematic; there will still be unmeasured or unobserved characteristics about those who agree to participate and thus who they represent. If convenience samples are used, the results should be interpreted carefully, and we highly recommend using other data in complementary analyses to help triangulate findings or provide additional context.

Efforts to seek funding might be better spent on value added measures such as anthropometry, biomarkers or nutrition label data on products. In other words, for primary data collection (whether quantitative or qualitative), researchers and funders should focus efforts and resources towards subpopulations, dietary segments or outcomes that are otherwise not available in secondary data and would clearly add value. Researchers may need to prioritise scientifically on the basis of availability and costs of data.

Researchers also need to determine if they should design their study as a cohort vs a repeat cross-section or other design and determine the implications of the options for causal inference. In some context, cohort studies need to be overpowered at baseline and can be challenging and expensive to maintain and track over time (e.g., in high migration areas) and are likely to have attrition bias that needs correction, and thus a repeated cross-sectional design may be more practical. Interrupted time series data, if available, is powered statistically on the number of time points rather than ‘population’ size, so can overcome the challenges of other designs.

For primary data that focuses on changes on the supply-side, such as changes in the pricing or formulation of products, there may be both more conventional or new approaches for collecting such information. For example, to obtain prospective nutrition label information, the team evaluating South Africa’s Health Promotion Levy collected beverage label data which was augmented by secondary nutrition label data[1]. Meanwhile, the UK SDIL evaluation team created a database of product formulations, prices and volumes (‘FoodDB’) by pulling information from the internet on a weekly basis.[2]

Of course, regardless of the design, care should be taken to ensure that the sample size and power, given multiple outcome measures of interest, are adequate. This is especially the case when certain subpopulations of interest are expected or hypothesized to respond differently as this would have implications for whether to oversample certain subgroups.

1. **Potential Secondary Data Sources**

Likewise, secondary data sources can also be both qualitative and quantitative. For qualitative analyses, documentary sources can be critical and include:

- Public and trade press (from databases such as Factiva and ProQuest).
- Government records (transcripts relating to policy process, consultations, implementation, etc.) including via Freedom of Information requests.
- Industry documents if publicly available (e.g., shareholder reports for publicly traded companies), leaked emails or through market research companies. Freedom of Information or legal discovery requests may be needed.
- Social media discourse (Twitter, Facebook, etc.) among public, government, advocacy groups and industry.

Government collected household or individual level data can be extremely useful sources of secondary data as they are often publicly available, designed to be nationally (sometimes regionally or subpopulation) representative, allows for easier replicability, and in certain countries routinely collected. However, the timing of when these data are made publicly available often means significant time lags between policy implementation and when evaluation of the policy using such data is conducted, peer-reviewed, published and used as evidence. These include:

- Micro-level price data: Government statistical offices often collect a representative sample of prices for a basket of goods (including foods and beverages) to construct consumer price indices. For example, Mexico’s National Institute of Statistics and Geography collects price data from 46 cities (above 20,000 inhabitants) covering the 32 states in the country and including the 10 most population urban areas. Rural areas are often not covered.
- National health and nutrition surveys (e.g., UK’s National Diet and Nutrition Survey) that include individual self-report measures of dietary intake: Ideally these would be based on at least two 24-hr recalls (with accompanying country-specific food composition tables), but typically these have been based on food frequency questionnaires. Many studies have discussed the pros and cons of various self-reported dietary intake assessments and researchers should keep these in mind when considering the specific setting where their evaluation is occurring.[3] Often, these are designed to be cross-sectional studies and if repeated regularly, provide surveillance on the nutritional wellbeing of the population over time and how these outcomes may differ across socio-demographic characteristics. Sometimes these surveys include objective measures of anthropometry and biomarkers for a subset of the sample. A key issue for repeated waves to be collected during the same seasons over time to ensure comparability in diets and diet-sensitive biomarkers.
- National household income and expenditure, household budget or standard of living surveys that include household self-report measures of food and beverages acquired over a period of time (e.g., one week): These surveys are designed mainly to evaluate financial and living conditions of households, rather than for nutrition or food habits specifically, and asks about purchases or acquisition, not consumption (does not account for food waste or loss, and purchases away from home may not have enough detail on type of food and beverage purchased). Moreover, the measurements are at the household level rather than at the individual level, and there are validated methods to translate these into adult male equivalent or the per capita amounts.[4]
- Other samples that represent specific populations of interest such as pre-schoolers, under-represented groups.

Other government sources of data on national, state or local statistics for inclusion that often released routinely include:

- National statistics on the amounts or value of import-export trade: typically at the monthly or quarterly basis and are organized based on Country and Standard International Trade Classification codes (SITC).
- Industry specific data on manufacturing amounts or value: often at the monthly or quarterly basis and are organized based on International Standard Industrial Classification for All Economic Activities Codes (ISIC).
- Industry or commercial establishment-specific employment statistics: often at the monthly or quarterly basis and are organized based on ISIC.[5, 6]
- Revenue generation and allocation statistics from various sources: Many tax collecting administrators are required to publicly report tax revenue collection amounts on a regular basis. In some cases when there are strong mechanisms and governance structure on tracking the revenue allocations, such data may also become public.

Commercial secondary data sources are also increasingly being used by health and food policy researchers and policy analysts. For example, Euromonitor provides annual aggregate national level on sales of “off-trade” and “on-trade” food and beverages for many countries[7, 8]. For some countries, Euromonitor also has nutrition information on top-selling brands going back to 2009. There are some costs associated with licensing this data. Planet Retail and Mintel are others that may also provide aggregate level data for assessing trends and allow for cross-country comparisons. Another creative source of business/finance data is to track stock returns of beverage companies in relation to key events (e.g., announcement and/or implementation of the tax) to assess if claims that such policies may have large-scale or long-lasting impacts on businesses has also been undertaken.[9, 10]

Other commercial data that are often expensive and difficult to obtain include retailer sales data, household level purchase data, and individual consumption data. Retailer sales data take a few forms, such as computerized point-of-sales data directly obtained from retail chains (e.g., as used in an early Berkeley study[11] and in the Barbados evaluation study[12]), and market research company collated data across retail chains on sales of items (e.g., Nielsen Scantrack, Kantar Retail).[13] Commercial household purchase data from market research companies like Nielsen, Kantar and GfK have been used in evaluations in several countries,[14-18] and include measurements of household characteristics (e.g., size, income or socio-economic status, head of household education, location) to help determine if there are heterogenous responses in demand by these characteristics. Some market research companies also conduct their own surveys on consumption behaviours, like the National Eating Trends Survey (NETS) by the NDP Group in the US. Often the costs of these commercial market data become more affordable the further the data licensing occurs as the “value” of these data depreciate given how reflective the data over time. Researchers could consider whether to wait to obtain such data depending on funding and timing constraints.

Earlier studies have assessed the strengths and weaknesses of public and commercial data sources for monitoring changes in diets and food purchases[19, 20] and have noted that commercial data sources are often timelier given the policy implications and relevance of the research, and objective measures based on sales are less likely to be subject to self-reporting bias. However, they are magnitudes more expensive not just due to the cost of licensing the data, but also because of the investments needed in establishing a system to work with these large commercial datasets. To date, evaluations using sales and purchases as outcomes have generally found larger changes compared to consumption/dietary intake measures. Sales data are likely to be more objectively captured compared to self-report intake which may be subject to mismeasurement due to recall challenges and/or social desirability bias. Moreover, the smaller sample sizes of current studies measuring consumption compared to purchase data means it will also be more difficult to detect statistical significance.

When the tax policy is based on sugar content and/or sweetener type, data on nutrition labels with information on nutrient levels, ingredients, package sizes, product descriptions and barcodes may be needed. In some countries, secondary data may already exist and should be explored. Examples include Label Insight (US) and FoodSwitch managed by The George Institute (Australia, New Zealand, China, India, South Africa), BrandView (UK) and Mintel (Global New Product Database). This could be particularly important if retrospective nutrition label data is needed.

Electronic health or medical records (EHR/EMR) might be the next frontier of big data in these evaluations. These may be worth exploring when there are measurable and meaningful changes in some of the more immediate measures in terms of calorie or sugar intakes. In countries where harmonised health and dental informatics systems are already established, this would be more possible to use for assessing potential health implications of policies.

Additional measures for inclusion in evaluation analyses may come from auxiliary datasets such as:

- Economic measures such as inflation rates, minimum wage, unemployment, gross domestic product per capita;
- Weather data such as temperatures (highs, lows), precipitation, humidity;
- Details of calendar events that influence consumption such as holidays or special events.

**REFERENCES**

1. Stacey N, Edoka I, Hofman K, Swart EC, Popkin B, Ng SW: **Changes in beverage purchases following the announcement and implementation of South Africa's Health Promotion Levy: an observational study**. *The Lancet Planetary Health* 2021, **5**(4):e200-e208.

2. Harrington RA, Adhikari V, Rayner M, Scarborough P: **Nutrient composition databases in the age of big data: foodDB, a comprehensive, real-time database infrastructure**. *BMJ Open* 2019, **9**(6):e026652.

3. Kirkpatrick SI, Reedy J, Butler EN, Dodd KW, Subar AF, Thompson FE, McKinnon RA: **Dietary Assessment in Food Environment Research: A Systematic Review**. *American Journal of Preventive Medicine* 2014, **46**(1):94-102.

4. Fiedler JL, Lividini K, Bermudez OI, Smitz M-F: **Household Consumption and Expenditures Surveys (HCES): A Primer for Food and Nutrition Analysts in Low- and Middle-Income Countries**. *Food and Nutrition Bulletin* 2012, **33**(3_suppl2):S170-S184.

5. Lawman HG, Bleich SN, Yan J, LeVasseur MT, Mitra N, Roberto CA: **Unemployment claims in Philadelphia one year after implementation of the sweetened beverage tax**. *PLOS ONE* 2019, **14**(3):e0213218.

6. Guerrero-López CM, Molina M, Colchero MA: **Employment changes associated with the introduction of taxes on sugar-sweetened beverages and nonessential energy-dense food in Mexico**. *Preventive medicine* 2017, **105**:S43-S49.

7. Bandy L, Adhikari V, Jebb S, Rayner M: **The use of commercial food purchase data for public health nutrition research: A systematic review**. *PLOS ONE* 2019, **14**(1):e0210192.

8. Baker P, Friel S: **Food systems transformations, ultra-processed food markets and the nutrition transition in Asia**. *Globalization and Health* 2016, **12**(1):80.

9. Law C, Cornelsen L, Adams J, Pell D, Rutter H, White M, Smith R: **The impact of UK soft drinks industry levy on manufacturers’ domestic turnover**. *Economics & Human Biology* 2020, **37**:100866.

10. Law C, Cornelsen L, Adams J, Penney T, Rutter H, White M, Smith R: **An analysis of the stock market reaction to the announcements of the UK Soft Drinks Industry Levy**. *Economics & Human Biology* 2020:100834.

11. Silver LD, Ng SW, Ryan-Ibarra S, Taillie LS, Induni M, Miles DR, Poti JM, Popkin BM: **Changes in prices, sales, consumer spending, and beverage consumption one year after a tax on sugar-sweetened beverages in Berkeley, California, US: A before-and-after study**. *PLoS Medicine* 2017, **14**(4):e1002283.

12. Alvarado M, Kostova D, Suhrcke M, Hambleton I, Hassell T, Samuels TA, Adams J, Unwin N: **Trends in beverage prices following the introduction of a tax on sugar-sweetened beverages in Barbados**. *Preventive medicine* 2017, **105**:S23-S25.

13. Roberto CA, Lawman HG, LeVasseur MT, Mitra N, Peterhans A, Herring B, Bleich SN: **Association of a Beverage Tax on Sugar-Sweetened and Artificially Sweetened Beverages With Changes in Beverage Prices and Sales at Chain Retailers in a Large Urban Setting**. *JAMA* 2019, **321**(18):1799-1810.

14. Colchero-Aragonés A, Rivera JA, Popkin B, Ng SW: **In Mexico, Evidence Of Sustained Consumer Response Two Years After Implementing A Sugar-Sweetened Beverage Tax**. *Health Affairs* 2017, **36**(3):564-571.

15. Caro JC, Corvalán C, Reyes M, Silva A, Popkin B, Taillie LS: **Chile’s 2014 sugar-sweetened beverage tax and changes in prices and purchases of sugar-sweetened beverages: An observational study in an urban environment**. *PLOS Medicine* 2018, **15**(7):e1002597.

16. Bandy LK, Scarborough P, Harrington RA, Rayner M, Jebb SA: **Reductions in sugar sales from soft drinks in the UK from 2015 to 2018**. *BMC Medicine* 2020, **18**(1):20.

17. Scarborough P, Adhikari V, Harrington RA, Elhussein A, Briggs ADM, Rayner M, Cummins S, Penney T, White M: **Impact of the announcement and implementation of the UK Soft Drinks Industry Levy on sugar content, price, product size and number of available soft drinks in the UK, 2015-19: controlled interrupted time series analysis**. *PLoS Med* 2020.

18. Pell D, Mytton O, Penney TL, Briggs A, Cummins S, Penn-Jones C, Rayner M, Rutter H, Scarborough P, Sharp SJ *et al*: **Changes in soft drinks purchased by British households associated with the UK soft drinks industry levy: controlled interrupted time series analysis**. *BMJ* 2021, **372**:n254.

19. Ng SW, Popkin BM: **Monitoring foods and nutrients sold and consumed in the United States: dynamics and challenges**. *Journal of the Academy of Nutrition and Dietetics* 2012, **112**(1):41-45 e44.

20. Ng SW, Dunford E: **Complexities and opportunities in monitoring and evaluating US and global changes by the food industry**. *Obesity Reviews* 2013, **14**(S2):29-41.
